# Advanced non-small cell lung cancer in patients aged 45 years or younger: outcomes and prognostic factors

**DOI:** 10.1186/1471-2407-12-241

**Published:** 2012-06-13

**Authors:** Chia-Lin Hsu, Kuan-Yu Chen, Jin-Yuan Shih, Chao-Chi Ho, Chih-Hsin Yang, Chong-Jen Yu, Pan-Chyr Yang

**Affiliations:** 1 Division of Pulmonary Medicine, Department of Internal Medicine, National Taiwan University Hospital, and College of Medicine, Taipei, Taiwan; 2 Department of Oncology, National Taiwan University Hospital, and College of Medicine, Taipei, Taiwan

**Keywords:** Lung cancer, Prognosis, Young patients

## Abstract

**Background:**

Lung cancer in young patients (less or equal to 45 years) is uncommon and has clinical characteristics different from that in older patients. We investigated the outcomes and prognostic factors of young patients with advanced non-small cell lung cancer (NSCLC).

**Methods:**

From January 2000 to December 2009, we enrolled patients aged ≤45 years and diagnosed with stage IIIB or IV NSCLC. Their clinical data, including age, gender, performance status, histologic types, disease stages, laboratory data at diagnosis, treatment modalities, and survival were reviewed and analyzed. A Cox proportional hazard model was used to calculate the hazard ratio (HR) and its 95% confidence interval (CI).

**Results:**

A total of 144 patients with advanced NSCLC were included. Female patients were more prevalent (n = 74, 51.4%). Adenocarcinoma was the most common histologic type (n = 119, 82.6%) in both genders (male, n = 54, 77.1%; female, n = 65, 87.8%). Epidermal growth factor receptor (*EGFR)* sequences were determined using tumor specimens from 58 patients, and 29 showed an *EGFR* mutation. No significant difference in median survival was found between patient groups with and without the *EGFR* mutation (798 vs. 708 days, *p* = 0.65). In multivariate analysis, male gender (HR, 1.70; 95% CI: 1.08-2.68), body mass index (BMI) less than 25 kg/m^2^ (HR, 2.72; 95% CI: 1.39-5.30), stage IV disease (HR, 2.62; 95% CI: 1.50-4.57), and anemia (HR, 2.08; 95% CI: 1.15-3.77) were associated with a short survival time.

**Conclusions:**

Low BMI, stage IV disease, anemia at diagnosis, and male gender were the negative prognostic factors for young patients with advanced NSCLC.

## Background

Lung cancer is the leading cause of cancer mortality worldwide, and has led to a number of important public health problems[1,2]. Despite advances in treatment, including surgery, chemotherapy, radiotherapy, and epidermal growth factor receptor (EGFR) tyrosine kinase inhibitor (TKI) therapy, the 5-year survival rate is only 9% to 20% [2,3]. Lung cancer in young adults is relatively rare [4-7]. The incidence of lung cancer among young adults has been found to be around 1.2% to 6.2% (under 40 years) [5-7], 5.3% (under than 45 years) [4], and 13.4% (under 50 years) [8]. However, previous reports have shown trends of increasing incidence rates of lung cancer among young patients [9,10].

Lung cancer in young adults has several unique characteristics: a high percentage of patients are female, more adenocarcinoma, more advanced stage at the time of diagnosis, and more patients receiving aggressive treatment [11-14]. Previous studies have shown that young patients had a similar survival as older patients [8,13,15]. In contrast, some studies have reported that younger patients have better outcomes [5,16,17]. Bourke *et al.* conducted a retrospective study of lung cancer patients under 45 years in Chicago and Israel [14]. The results showed that younger patients had shorter survival than older patients in Chicago, but longer survival in Israel. A recent multicenter study in China showed that middle-aged patients had longer median survival than younger patients [4]. These inconsistent findings could be related to different treatment modalities and ethnicities of patients.

Little has been studied about the prognostic factors for young patients with lung cancer. A retrospective study analyzing young patients (n = 91) with all disease stages from 1983 to 1993, reported that disease stages and weight loss affected survival [13]. Because of the paucity of data for prognosis, we conducted a retrospective study to investigate the outcomes and prognostic factors in young patients (≤45 years) with advanced non-small cell lung cancer (NSCLC). We also reviewed available data on *EGFR* mutation in the tumor tissues of young patients and analyzed the relationship between *EGFR* mutation and EGFR-TKI treatment efficacy.

## Methods

### Patients

Patients with a diagnosis of NSCLC between January 2000 and December 2009 were identified by using the International Classification of Diseases, Version 9 coding system from a computer registration database in the National Taiwan University Hospital. Those patients aged 45 years or younger and at an advanced stage of disease, defined as stage IIIB or IV, were included. The diagnosis was based on pathology via surgical or biopsy specimens, or cytology examination via lung, lymph node aspiration or pleural effusion. Exclusion criteria included the clinical diagnosis of lung cancer without proven pathology or cytology, or cases in which the lung cancer was combined with another type of malignancy. This study was approved by the Institutional Review Board of the National Taiwan University Hospital.

### Data collection

The clinical data, including age, gender, initial presentation, performance status (PS), disease stages, laboratory data at diagnosis, treatment modality and responses, progression-free survival (PFS) for initial treatment, and overall survival (OS) were recorded. The disease stage was determined by surgical or clinical findings according to the TNM classification system [18]. Eastern Cooperative Oncology Group (ECOG) PS was determined according to the records for the patients’ activity of daily life and the extent of dependence. Anemia was defined as hemoglobin of less than 11 g/dL; leukocytosis, white cell count over 10,000/μL; thrombocytosis, platelet count over 400,000/μL; hypoalbuminemia, serum albumin level of less than 3.5 g/dL, and abnormal liver function, aspartate aminotransferase (AST) level over 37 U/L or alanine aminotransferase (ALT) level over 41 U/L.

Whenever available, we analyzed *EGFR* mutation status using a QIAmp DNA Mini kit (Qiagen, Valencia, CA) on DNA extracted from lung cancer specimens embedded in paraffin blocks. *EGFR* exons 18, 19, 20, and 21 were amplified by using nested polymerase chain reaction (PCR). PCR conditions have been described previously [19].

### Statistical analysis

Patients’ characteristics were compared using the chi-square test. Continuous variables were compared by using an independent *t*-test. PFS was defined as the duration from the date of initial treatment to the date of clinical deterioration that was described in the medical records. OS was defined as the time from the date of diagnosis to the date of death or last visit. The patients were followed up until March 31, 2011 or until the date of their death. PFS and OS were analyzed by using the Kaplan-Meier method with log-rank test.

We conducted univariate analyses to examine the unadjusted associations between patient groups with each independent variable. Age, gender, and factors associated with patient survival in univariate analyses with a *p* value < 0.10 were included. Proportional hazard assumption was tested via extended Cox regression model with time-dependent covariate and log-minus-log plot. Multicollinearity diagnostics were also performed. Multivariate analysis with Cox proportional hazard model was applied to calculate the hazard ratio (HR) and its 95% confidence interval (CI). Data were analyzed using SPSS software (version 18).

## Results

### Clinical characteristics

**Figure 1:**
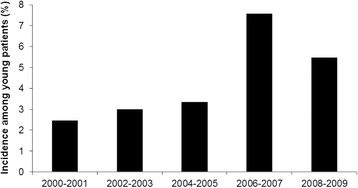
Incidence of non-small cell lung cancer among young patients between 2000 and 2009.

Between January 2000 and December 2009, 5,871 patients were diagnosed with lung cancer at the National Taiwan University Hospital. A total of 225 patients (3.8%) were 45 years or younger. There was a trend of increasing incidence of lung cancer among young patients during this period (Figure 1). Among them, 64 patients had stage I to IIIA NSCLC, 5 had small cell lung cancer, and 12 did not continue follow-up after the initial diagnosis. In the end, 144 patients with stage IIIB or IV NSCLC were included for analysis.

The clinical characteristics of these patients are outlined in Table 1. Their median age was 39.1 years and 51.4% were women. The most common initial clinical presentation was cough (54.2%), followed by dyspnea (19.4%) and chest pain/tightness (17.4%). Six patients (4.2%) were asymptomatic, with abnormal chest image findings during health examination. There were 62 patients (43.1%) with a history of cigarette smoking. Fifty (34.7%) were current smokers. Most patients (n = 106, 73.6%) had a PS of 0 to 1.

**Table 1:** The clinical characteristics of 144 young patients with advanced non-small cell lung cancer

**Clinical characteristics**	**n(%)**
Age, median (range)	39.1 (22–45)
BMI (kg/m^2^)	22.1 ± 3.3
Male gender	70 (48.6%)
Smoking (current or ex-smoker)	62 (43.1%)
**Initial presentations**	
Pulmonary	
cough	78 (54.2%)
dyspnea	28 (19.4%)
chest pain/tightness	25 (17.4%)
hemoptysis	18 (12.5%)
hoarseness	4 (2.7%)
Extrapulmonary	
bone/muscle pain	15 (10.4%)
subcutaneous mass/lymphadenopathy	7 (4.9%)
fever	7 (4.9%)
focal neurological signs	6 (4.2%)
headache	5 (3.5%)
body weight loss	5 (3.5%)
others	8 (5.6%)
health exam	6 (4.2%)
**Performance status (PS)**	
0-1	106 (73.6%)
2-4	38 (26.4%)
**Disease stage**	
IIIB	40 (27.8%)
IV	104 (72.2%)
**Histology**	
Adenocarcinoma	119 (82.6%)
Squamous cell carcinoma	9 (6.3%)
Undifferentiated carcinoma	16 (11.1%)

BMI: body mass index.

**Figure 2:**
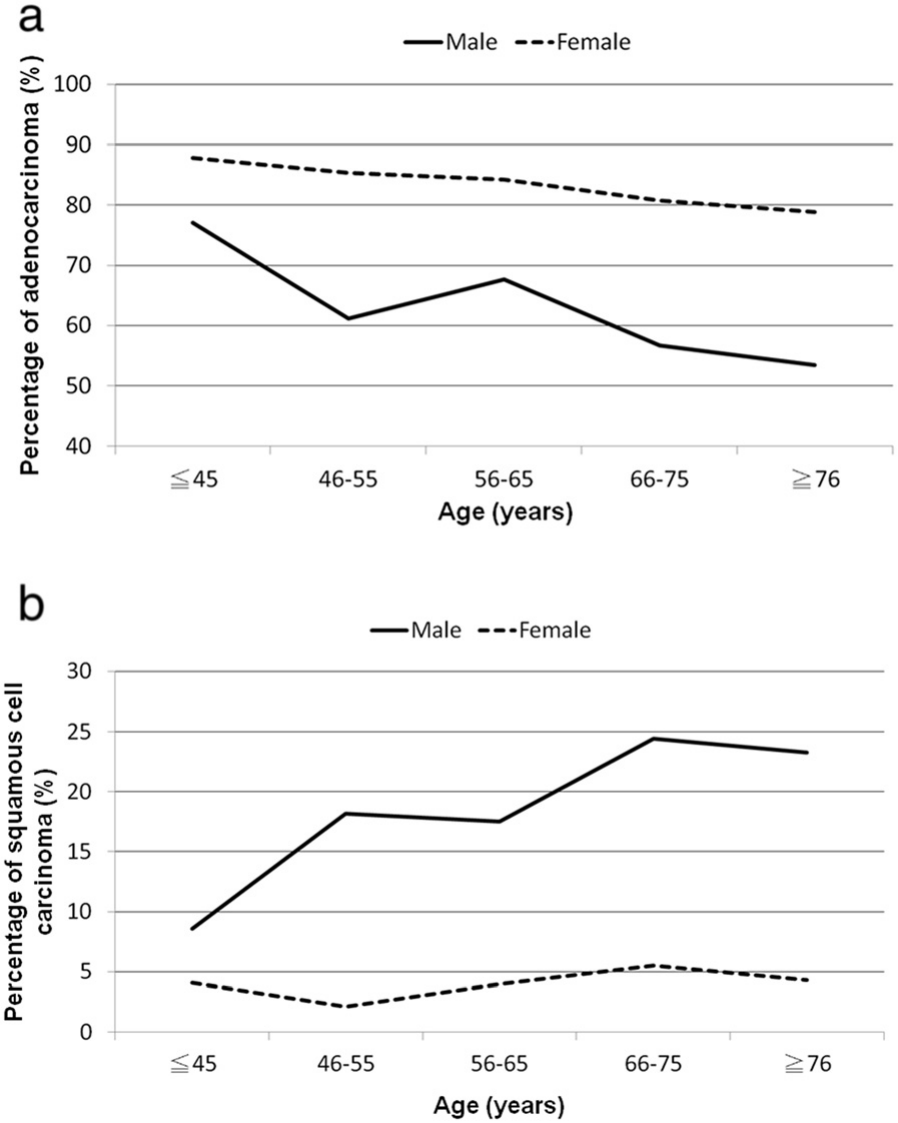
**a. Proportion of adenocarcinoma in different age groups.b**. Proportion of squamous cell carcinoma in different age groups.

Adenocarcinoma was the most common histologic type (n = 119, 82.6%), followed by undifferentiated carcinoma (n = 16, 11.1%) and squamous cell carcinoma (n = 9, 6.3%). There were no differences in the proportion of adenocarcinoma between genders (female vs. male, 87.8% vs. 77.1%, *p* = 0.12). Compare to other age groups (46–55, 56–65, 66–75, and 76 years or older) on the registry database of the National Taiwan University Hospital, younger patients (≤45 years) of both genders had the highest proportion of adenocarcinoma, and young males had the lowest proportion of squamous cell carcinoma (Figure 2a and 2b). Regarding disease stage, 40 patients (27.8%) had stage IIIB disease and 104 (72.2%) had stage IV disease. There were no significant differences between male and female patients in stage distribution.

### Treatment modalities and response

For initial treatment, 85 patients (59.0%) received chemotherapy and 41 patients (28.5%) received EGFR-TKI therapy. Five patients (3.5%) who received concomitant chemotherapy and EGFR-TKI were classified as receiving other treatment. Thirteen patients (9.0%) received supportive care. No significant differences were found between patient groups with PS 0 to 1 and PS 2 to 4, with regard to treatment modalities.

Among the patients who received chemotherapy, 4 patients could not be evaluated for treatment response, including 3 patients who lost during follow-up and 1 who discontinued chemotherapy because of drug toxicity. The treatment responses of the 81 patients included 36 with partial response (44.4%), 31 with stable disease (38.3%), and 14 with progressive disease (17.3%). Among the patients who received EGFR-TKI as their initial treatment, 2 patients were lost to follow-up during treatment. The treatment responses of the remaining 39 patients included 16 with partial response (41.0%), 15 with stable disease (38.5%), and 8 with progressive disease (20.5%).

### Survival and prognostic factors

There were 125 deaths during the follow-up period. The median survival was 14.7 months. According to the registry database of the National Taiwan University Hospital from 2004 to 2008, the median survival of advanced NSCLC patients in the age groups 46 to 55 (n = 362), 56 to 65 (n = 511), 66 to 75 (n = 622) and over 76 years (n = 499) were 19.7, 15.0, 13.0, and 7.0 months, respectively. The median survival of patients ≤45 years old was significantly shorter than that of patients aged 46 to 55 years (*p* = 0.02) and longer than that of patients ≥76 years old (*p* < 0.001). No significant difference was found when the median survival of young patients was compared to that of the patients aged 56 to 65 and 66 to 75 years.

To compare the effects of major treatments (chemotherapy vs. EGFR-TKI), we excluded patients who received supportive treatment (n = 13) and concomitant chemotherapy and EGFR-TKI (n = 5) for prognostic factor analysis. A total of 126 patients were included in prognostic factor analysis. In univariate analysis, body mass index (BMI) of less than 25 kg/m^2^, stage IV disease, poor PS, anemia, leukocytosis, thrombocytosis, and hypoalbuminemia were related to poor outcomes (Table 2). We examined the proportional hazard assumption and multicollinearity, and neither assumption violation nor significant multicollinearity was noted. In multivariate analysis, we found that male gender (HR, 1.70; 95% CI: 1.08-2.68), BMI of less than 25 kg/m^2^ (HR, 2.72; 95% CI: 1.39-5.30), stage IV disease (HR, 2.62; 95% CI: 1.50-4.57), and anemia (HR, 2.08; 95% CI: 1.15-3.77) were associated with a poor prognosis (Table 3).

**Table 2:** Univariate prognostic factor analysis of advanced non-small cell lung cancer in young adults

	**Hazard Ratio (95% confidence interval)**	***p* value**
Age > 40 years	1.00 (0.67–1.48)	0.99
Male gender	1.25 (0.85–1.82)	0.26
BMI < 25 kg/m^2^	2.82 (1.52–5.26)	0.001
Smoking history	0.96 (0.65–1.41)	0.83
Medical co-morbidity	1.26 (0.55–2.89)	0.58
**Clinical stage**		
IIIB	1	
IV	2.24 (1.43–3.49)	<0.001
**Performance status**		
0-1	1	
2-4	1.59 (1.04–2.43)	0.03
**Histology**		
Adenocarcinoma	1	
Squamous cell carcinoma	1.02 (0.41–2.52)	0.97
Undifferentiated	1.26 (0.67–2.37)	0.47
**Laboratory data at diagnosis**		
Anemia (hemoglobin < 11 g/dL)	2.44 (1.45–4.10)	0.001
Leukocytosis (>10,000 cells/μL)	2.72 (1.75–4.21)	<0.001
Thrombocytosis (>400,000 cells/μL)	1.70 (1.00–2.90)	0.05
Hypoalbuminemia (<3.5 g/dL)	3.26 (1.69–6.30)	<0.001
Abnormal liver function	1.12 (0.68–1.83)	0.65
**Initial treatment modalities**		
Chemotherapy	1	
EGFR-TKI therapy	1.14 (0.75–1.71)	0.54

BMI: body mass index; EGFR-TKI: epidermal growth factor receptor-tyrosine kinase inhibitor.

**Table 3:** Multivariate analysis of prognostic factors in young advanced NSCLC

	**Hazard Ratio (95% confidence interval)**	***p* value**
Male gender	1.70 (1.08–2.68)	0.02
BMI < 25 kg/m^2^	2.72 (1.39–5.30)	0.003
**Disease stage**		
IIIB	1	
IV	2.62 (1.50–4.57)	0.001
**Performance status**		
0-1	1	
2-4	1.59 (0.96–2.63)	0.08
**Laboratory data**		
Anemia (hemoglobin < 11 g/dL)	2.08 (1.15–3.77)	0.02
Leukocytosis (> 10,000 cells/μL)	1.62 (0.94–2.78)	0.08

BMI: body mass index.

### EGFR mutation and prognosis

Fifty-eight patients received examination for *EGFR* mutations, and 29 patients (50.0%) had *EGFR* mutations, including 11 with L858R, 1 with L858R + T790M, 1 with G719A + T790M, 1 with R776C, 1 with S768-D770 dup, and 14 with exon 19 deletions. Female patients and non-smokers had higher rates of *EGFR* mutations (59.4% vs. 38.5%, *p* = 0.09; and 60.0% vs. 30.8%, *p* = 0.05, respectively), but the difference was not statistically significant. The median survival was similar in patients with and without *EGFR* mutations (798 vs. 708 days, *p* = 0.65).

**Figure 3:**
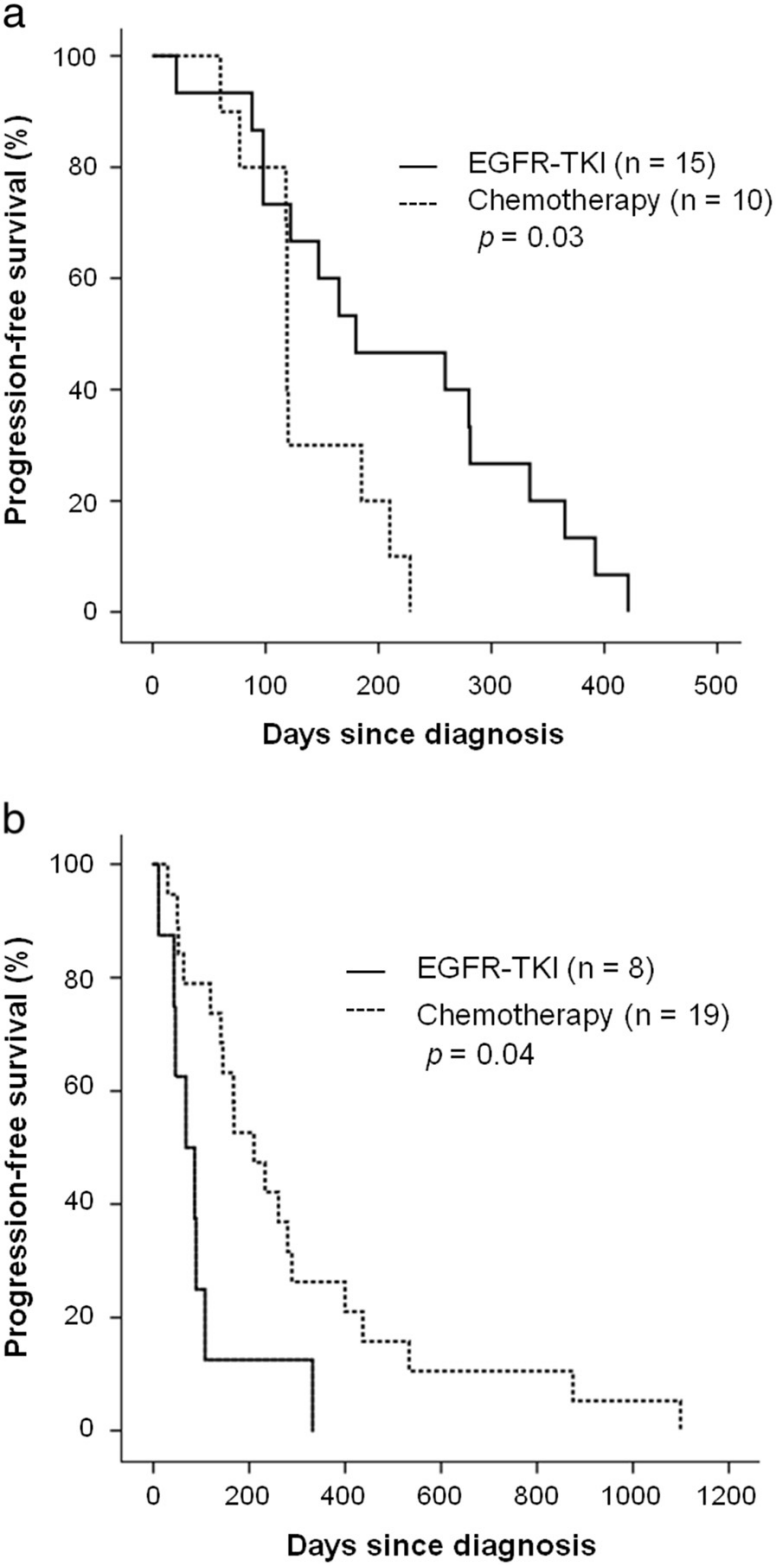
**a. Progression-free survival after chemotherapy and epidermal growth factor receptor-tyrosine kinase inhibitor (EGFR-TKI) therapy in patients with the*EGFR*mutation.b.** Progression-free survival after chemotherapy and epidermal growth factor receptor-tyrosine kinase inhibitor (EGFR-TKI) therapy in patients without the *EGFR* mutation.

Of the 29 patients with *EGFR* mutations, 15 received EGFR-TKI as their initial treatment, 10 received chemotherapy, 3 received concomitant chemotherapy and EGFR-TKI, and 1 received supportive care. Median PFS was longer in patients who received EGFR-TKI as their initial treatment than in patients who received chemotherapy as their initial treatment (180 vs. 119 days, *p* = 0.03) (Figure 3a). However, there was no significant difference in OS between the 2 groups (EGFR-TKI vs. chemotherapy, 597 vs. 798 days, *p* = 0.89).

Of the 29 patients without *EGFR* mutations, 8 received EGFR-TKI, 19 received chemotherapy and 2 received only supportive care as their initial treatment. Median PFS and OS were shorter in patients who received EGFR-TKI as their initial treatment than in patients who initially received chemotherapy (68 vs. 210 days, *p* = 0.01; and 117 vs. 891 days, *p* = 0.04, respectively) (Figure 3b).

## Discussion

Our study demonstrated the clinical presentations, and *EGFR* mutation status, in young patients with advanced NSCLC, a relative rare patient population with many differences from the older patient population. We also found that low BMI, stage IV disease, anemia at diagnosis, and male gender were the negative prognostic factors, which seldom investigated in previous literatures. These findings may improve comprehension of this special patient population.

Younger patients had higher rate of adenocarcinoma (57.5-77.9%)[4-8,11]. Our study showed that adenocarcinoma was the predominant histologic type in young patients; of both genders. The reason for the extremely high percentage of adenocarcinoma in young patients has been seldom studied and requires more attention. As compared with older patients, a higher female percentage in young patients was presented in several studies (36.1-48.7%)[4,5,7]; however most studies have shown that males were the predominant gender, except for studies in Taiwan (51.8-52.5%) [6,11], as well as in our study (51.4%). This might be related to the ethnicities of patients and environmental effects [20].

The outcomes of young and old patients with lung cancer had been previously studied, but the results were inconsistent [5,8,13-17]. Most studies compared only the outcomes of younger and older patients, but not the outcomes of different age groups. Our study showed that the median OS of patients aged ≤45 years was significantly shorter than that of patients aged between 46 and 55 years and was longer than that of patients aged ≥76 years. No significant difference was found when we compared the median OS of young patients with the patients aged between 55 to 65 and 66 to 75 years. A similar result was obtained in a recent Chinese study.[4]. The explanation for the survival difference remains unclear.

The influence of BMI has been studied in several types of cancers. In gynecological malignancies, a high BMI is associated with a higher incidence of cancer but better disease-specific survival [21,22]. Similar results have also been found for gastric cancer and renal cell carcinoma [23,24]. However, high BMI is a poor prognostic factor in breast cancer [25-27]. Obese patients may have better nutritional resources to withstand the stress of cancer metastasis and cachexia [28].

Little is known about the association between prognosis and BMI in lung cancer. In epidemiology studies, obese patients had lower lung cancer mortality; however, the mechanism is unknown [29-31]. A recent study by Yang *et al*. showed that patients who were obese at the time of lung cancer diagnosis had a longer median survival for all cancer stages and histologic subtypes [32]. The obesity paradox is that being overweight increases the incidence of heart failure, but in patients with chronic heart failure, obesity is associated with better survival. This phenomenon appears to also apply to diseases with high catabolic states, such as chronic kidney disease, rheumatoid arthritis and lung cancer [32-34].

Hematologic abnormalities, including anemia, have been reported as prognostic factors in many solid tumors [35-38]. In our study, anemia was shown to be an independent negative prognostic factor. The incidence of anemia in lung cancer patients varies from 6.5% to 34% [38,39]. Lung cancer patients presenting with anemia have an increased 19% risk of death [40]. However, erythropoietin treatment of anemic cancer patients has failed to demonstrate survival benefits and may even harmful [41,42]. It should be noted that anemia could merely reflect the disease severity, rather than the direct cause of mortality [40,43].

The frequency of *EGFR* mutation in adenocarcinoma showed wide variation among different reports (3%-59.7%); this was related to ethnicity, gender, and smoking status [19,44-47]. Several studies, in which East Asia was the focus, have shown that the *EGFR* mutation rate in NSCLC was about 26% to 38.6%, and the mutation rate increased 32% to 55% in adenocarcinoma [19,44,45]. However, there is a paucity of data regarding the *EGFR* mutation status in young patients. Our study showed that the *EGFR* mutation rate was 50.0% in advanced cases of NSCLC and 51.8% in cases of adenocarcinoma. The mutation rate was relatively high; these results could be attributed to the high percentage of female patients and fewer numbers of smokers among young patients.

Of the patients with positive *EGFR* mutations, patients who received EGFR-TKI had a longer PFS as compared to patients who received chemotherapy. This result is compatible with the Iressa Pan-Asia study and a phase 3 clinical trial conducted by the North-East Japan Study Group [46,48]. However, the median PFS and survival time in the EGFR-TKI group of our study were only 6.0 and 19.9 months, respectively. This was clearly shorter than the corresponding values obtained in a phase 3 trial in Japan (PFS, 10.8 months; survival time, 30.5 months) [48]. Similar results with a shorter PFS were also observed for the chemotherapy group (this study vs. phase 3 trial in Japan, 4.0 vs. 5.4 months) [48]. This suggests that the young patients with advanced lung adenocarcinoma have a poorer treatment response compared to the general population, especially with regard to EGFR-TKI.

There are several limitations to this study. First, the study was retrospective in design and included a relatively small case number. Second, because only a relatively small number of patients received *EGFR* mutation analysis, the results of *EGFR* mutation and EGFR-TKI treatment in the young lung cancer patients should be interpreted carefully.

## Conclusions

Male gender, low BMI, late stage disease, and anemia were the negative prognostic factors for advanced NSCLC in young patients. More large scale studies on young NSCLC patients are warranted.

## Abbreviations

ALT, Alanine aminotransferase; AST, Aspartate aminotransferase; BMI, Body mass index; CI, Confidence interval; ECOG, Eastern Cooperative Oncology Group; EGFR, Epidermal growth factor receptor; HR, Hazard ratio; NSCLC, Non-small cell lung cancer; OS, Overall survival; PFS, Progression-free survival; PS, Performance status; TKI, Tyrosine kinase inhibitor.

## Competing interests

The authors declare that they have no competing interests.

## Authors’ contributions

CL Hsu contributed to manuscript draft, data collection and statistical analysis, KY Chen contributed to study design and statistical analysis, JY Shih, CC Ho and CH Yang participated in study design and data collection, CJ Yu and PC Yang contributed to manuscript revising. All authors read and approved the final manuscript.

## Pre-publication history

The pre-publication history for this paper can be accessed here:

http://www.biomedcentral.com/1471-2407/12/241/prepub
